# Prevalence and genotypes distribution of group A rotavirus among outpatient children under 5 years with acute diarrhea in Shanghai, China, 2012–2018

**DOI:** 10.1186/s12876-022-02288-9

**Published:** 2022-05-03

**Authors:** Lijuan Lu, Huaqing Zhong, Ran Jia, Liyun Su, Menghua Xu, Lingfeng Cao, Pengcheng Liu, Yuanyun Ao, Niuniu Dong, Jin Xu

**Affiliations:** 1 Department of Clinical Laboratory, Children’s Hospital of Fudan University, 399 Wanyuan Road, Shanghai, 201102 People’s Republic of China; 2 Department of Pediatric Institute, Children’s Hospital of Fudan University, Shanghai, 201102 People’s Republic of China

**Keywords:** Children, Diarrhea, Genotype, Outpatient, Rotavirus

## Abstract

**Background:**

Group A rotavirus (RVA) remains the main causative agent of acute diarrhea among children under five years in countries that have not yet introduced the RVA vaccine worldwide. Long-term and continuous monitoring data on RVA infection in outpatient children were lacking in Shanghai. We investigated the prevalence and distribution of RVA genotypes in outpatient children with acute diarrhea in Shanghai from 2012 to 2018.

**Methods:**

Stool specimens of outpatient children under five years were collected from the Children’s Hospital of Fudan University in Shanghai, China. All the samples enrolled in this study were detected and characterized for the P and G genotypes of RVA were determined using the semi-multiplex RT-PCR technique.

**Results:**

Of 1814 children enrolled with acute diarrhea and having specimens collected, 246 (13.6%) were infected with RVA. The highest frequency of RVA was observed in children younger than two years old (87.0%, 214/246). Year-round RVA transmission was observed and the RVA detection rate peaked every winter and troughed in summer. In this study, 12 different RVA strains were identified in children. G9P[8] (49.2%, 121/246) was detected as the most prevalent genotype, followed by G–P[8] (22.8%, 56/246), G3P[8] (11.4%, 28/246), and G9P- (4.9%, 12/246). Although RVA strains detected in this study varied with the time, G9P[8] has been the most predominant circulating genotype since 2012. Furthermore, 12.2% (30/246) RVA positive samples were co-infected with other diarrhea viruses.

**Conclusion:**

The present analysis showed that RVA was still a major cause of children with acute diarrhea in Shanghai from 2012 to 2018. A great diversity of RVA strains circulated in children with acute diarrhea with G9P[8] being the predominant genotype since 2012. Long-term and continuous monitoring of RVA genotypes is therefore indispensable to refine future vaccine strategy in Shanghai.

## Introduction

With the successful implementation of effective rotavirus vaccines worldwide, the death toll caused by rotavirus gastroenteritis dropped from 453,000 in 2008 to 128,000 children in 2016 according to the estimation of the World Health Organization (WHO) and the Global Burden of Disease (GBD) [1, 2]. However, group A rotavirus (RVA) remains the most common aetiological agent of diarrhea-associated morbidity and mortality in children less than 5 years of age, especially in the developing regions where rotavirus vaccines were unavailable [3–5]. Long-term surveillance for the distribution and genetic diversity of rotavirus is vitally important for vaccination policy in children under five years. Rotavirus is a non-enveloped virus and is classified as a member of the family Reoviridae. The genome consists of 11 segments of double-stranded RNA (dsRNA) which encodes six viral structural proteins (VP1, VP2, VP3, VP4, VP6, and VP7) and six non-structural proteins (NSP1, NSP2, NSP3, NSP4, NSP5, and NSP6). Encompassing the dsRNA are 3 protein layers, the core–shell is formed by VP2, the middle player is composed by VP6 and the outer capsid consists of VP7 and VP4 proteins [11]. RV is classified into nine recognized species (RVA-RVI) and a tentative species (RVJ) according to the antigenicity of VP6 protein. Among those, RVA is the most widespread species in humans [12–14]. Traditionally, binomial classification of RVA is adopted based on the nucleotide sequence of genomic segments of the VP7 (G genotypes) and VP4 (P genotypes) [15].

In China, a locally manufactured, live attenuated, oral, lamb rotavirus G10P[12] strain vaccine, Lanzhou Lamb RVA (LLR) vaccine (Lanzhou Institute of Biological Products, Lanzhou, China) has been available since 2000 [6, 7].This vaccine is currently not included in the Chinese Expanded Program on Immunization routine immunization schedule [8]. From 1998 to 2013, over 40% of diarrhea hospitalizations and ~ 30% of diarrhea-related outpatient visits in children aged < 5 years were caused by RVA in China [8]. In addition, the burden of RVA-associated diarrhea in China was enormous and estimated to cost about $61.4 million per year [9]. So far, rotavirus is still a leading cause of diarrheal disease among children aged < 5 years in China [3, 10]. To date, more than 41 G and 57 P genotypes have been detected in humans and other animals (https://rega.kuleuven.be/cev/viralmetagenomics/virus-classification). Globally, G1-G4, G9, G12 are the most common G types, whereas P [4] and P [8] are the most common P genotypes [9, 16]. Although more than 70 G and P genotype combinations have prevailed in humans and animals, G1P[8], G2P[4], G3P[8], G4P[8], and G9P[8] genotypes were the most common G-P genotype combinations in children infected with RVA through the worldwide [2, 17].

The distribution of RVA genotypes over the years in the same area is characterized by natural and cyclical genotype fluctuations [10, 18]. The new and dynamic epidemiological scenario reinforces the need to continuously document RVA prevalence in children with acute diarrhea. This study aimed to investigate the features of RVA prevalence and the molecular characterization of G and P genotypes among children with acute diarrhea in Shanghai from 2012 to 2018.

## Materials and methods

### Study population and fecal specimens

During January 2012 and December 2018, a total of 1814 stool specimens were collected from outpatient children aged ≤ 5 years and diagnosed with acute diarrhea in Children’s Hospital of Fudan University in Shanghai, China. Acute diarrhea was defined as over three soft or liquid stools with or without associated symptoms such as vomiting, fever, and abdominal pain [19]. Enrolled specimens were adopted by random sampling method from children diagnosed with acute diarrhea. The sample size enrolled in each year was 144, 144, 144, 265, 313, 423, and 381 cases from 2012 to 2018. Stool specimens enrolled in this study were stored at – 70 °C until further analysis. Demographic characteristics of children with acute diarrhea were collected from medical history by the professional involved. Informed consent was not required from the patients because the stool specimens were collected during the normal course of patient care. This study was approved by the Ethical Review Committee of Children's Hospital of Fudan University.

### Viral nucleic acid extraction

Stool suspensions were prepared as 10% (w/v) in saline solution. Total nucleic acid was extracted from 200 μL of suspensions using a TIANamp Virus DNA/RNA Kit (TIANGEN Biotech (Beijing) Co., Ltd.) according to the manufacturer’s instructions. The final viral genomes were eluted with 40 μL DEPC H_2_O and kept frozen at − 70 °C for further use.

### Semi-nested multiplex RT-PCR for RVA genotyping

All of the extracted genetic material were tested by semi-nested multiplex reverse transcription-polymerase chain reaction (RT-PCR) using type-specific primers for VP7-G1, G2, G3, G4, and G9 and for VP4-P[4], P[6], P[8], and P[9] [20]. In brief, the first amplification was performed using a one-step RT-PCR kit (Shanghai BoFeng Biotechnology Co., Ltd.) with specific primers for the identification of VP7 and VP4 regions respectively. Detailed PCR reaction process was as follows: after denaturation of the RNA at 97 °C for 5 min, the reaction were incubated on ice until reverse transcription; one-step RT-PCR was conducted under the following conditions: 30 min at 42 °C; 2 min at 95 °C; 45 cycles of 30 s at 94 °C, 30 s at 55 °C and 1 min at 72 °C; 7 min at 72 °C; and then 4 °C hold. The second amplification was conducted using the first PCR product as the template with G or P type-specific mixed primer to identify G or P genotypes. The amplification condition was 94 °C for 2 min, followed by 35 cycles at 94 °C for 30 s, 44 °C for 45 s, 72 °C for 1 min, and with an extension at 72 °C for 7 min, and then 4 °C hold. The PCR products were electrophoresed using 2% agarose gel, the G and P genotypes were determined according to their respective product size.

### Detection of HuCV, HAdV and HAstV

Viral genomic was reverse transcribed using PrimeScript™ II Reverse Transcriptase (Takara, Biotechnology (Dalian) Co., Ltd.) according to the manufacturer’s instruction. cDNA was amplified by PCR for the presence of human calicivirus (HuCV), and human astrovirus (HAstV). Human adenovirus (HAdV) was analyzed by PCR. Detailed instructions were conducted as described previously [20].

### Statistical analysis

The results were analyzed using IBM SPSS Statistics version 20.0 (IBM Corp. Armonk, New York, USA) software package. Statistical methods were applied according to the characteristics of the data in this study. Proportions for categorical variables were compared using the χ^2^ test. Two-sided *p*-values of less than 0.05 were considered statistically significant.

## Results

### Demographic characteristics of children

A total of 1814 stool samples were collected from symptomatic outpatients diagnosed with acute diarrhea from January 2012 to December 2018. All of the children enrolled in this study were younger than 5 years. Diarrhea cases were further distributed into the following five age groups: 0–6 months (n = 414; 22.8%), 7–12 months (n = 837; 46.1%), 13–24 months (n = 258; 14.2%), 25–36 months (n = 105; 5.8%) and 37–60 months (n = 200; 11.0%). Among the pediatric patients, 62.1% (1126/1814) were males and 37.9% (688/1814) were females.

### Prevalence of RVA in children

**Fig. 1:**
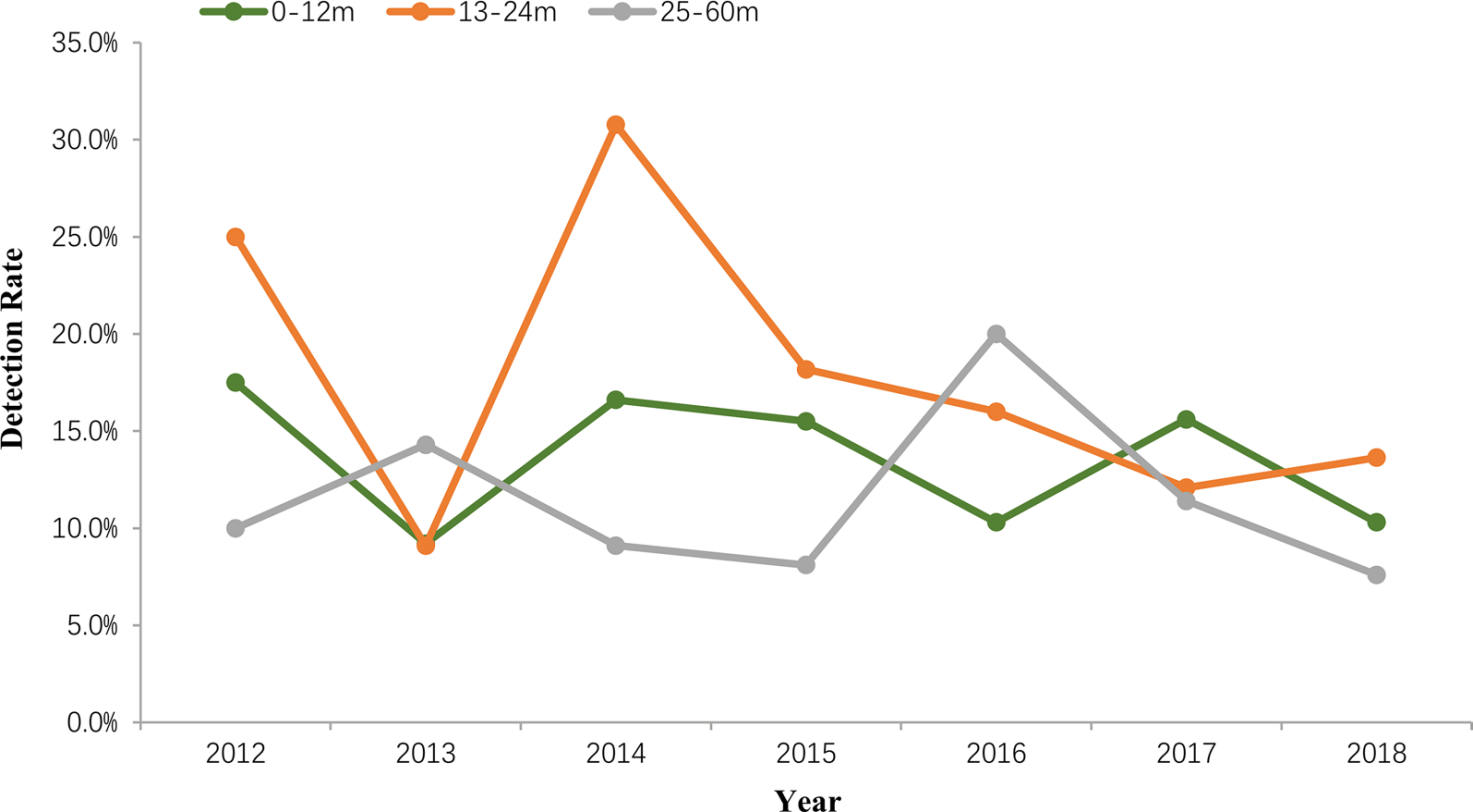
Age distribution of group A rotavirus (RVA) detected in outpatient children per year in Shanghai

**Fig. 2:**
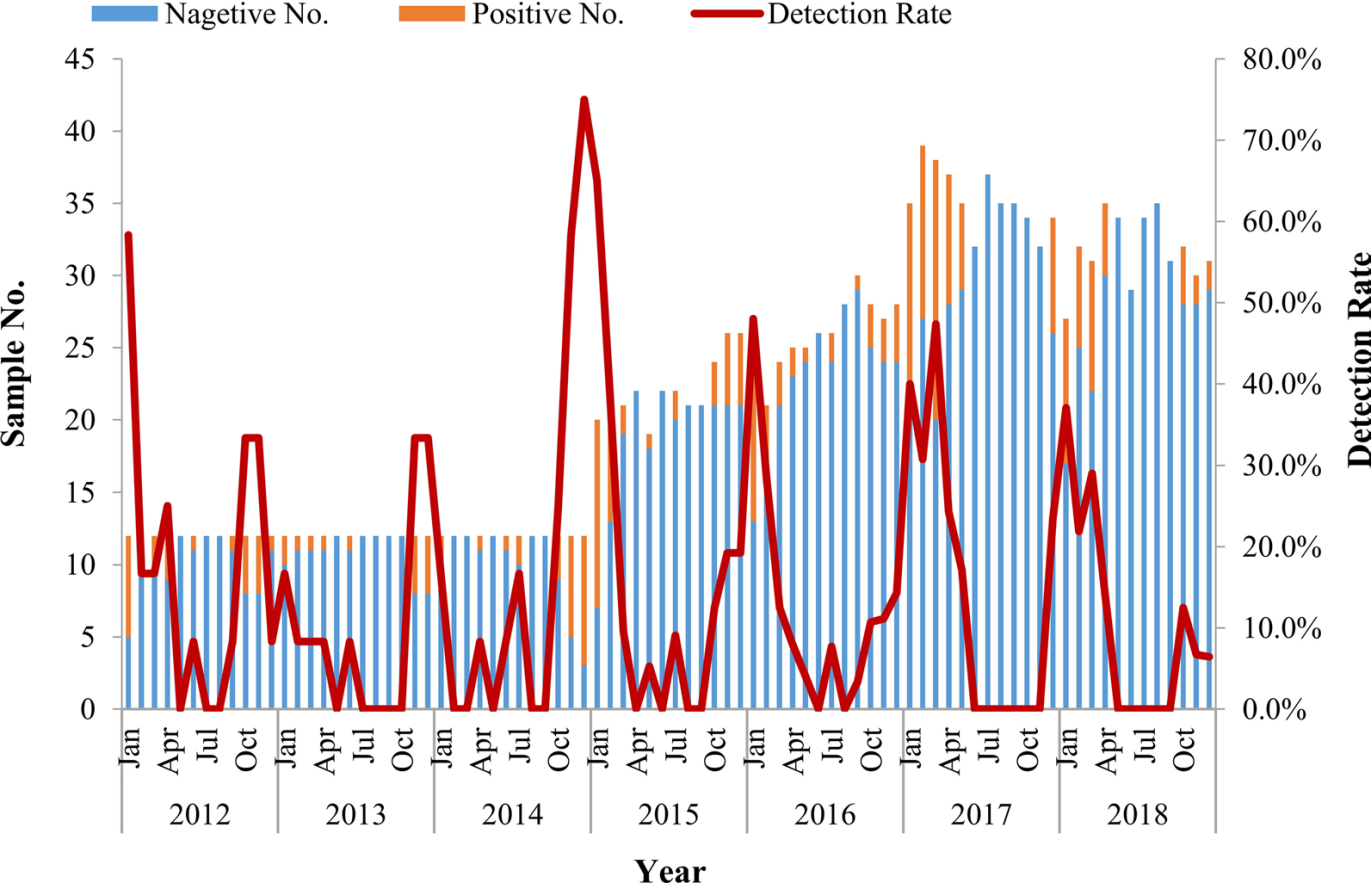
Monthly distribution of RVA detected in outpatient children in Shanghai

Overall, 13.6% (246/1814) samples were positive for RVA detected by semi-nested multiplex RT-PCR. From 2012 to 2018, the detection rate of RVA were 17.4% (25/144), 9.7% (14/144), 17.4% (25/144), 14.7% (39/265), 11.8% (37/313), 15.8% (67/423) and 10.2% (39/381), respectively. RVA infection was detected in all age groups with the most prevalence of RVA infection occurring in children aged from 7 to 12 months (16.3%, 136/837), followed by children in the age group of 25 to 36 months (16.2%, 17/105) and 13 to 24 months (14.7%, 38/258). The detection rate of RVA in children distributed to the same age group was varied during 2012 and 2018 (Fig. 1). A similar frequency of RVA was observed in both males (13.3%, 150/1126) and females (14.0%, 96/688) diarrhea cases (χ^2^ = 0.146, *P* = 0.703). In this study, the seasonal distribution of RVA had an obvious seasonal pattern. RVA positive detection rate peaked every winter and trough in summer from 2012 to 2018 (Fig. 2).

### Distribution of G and P genotypes of RVA

**Fig. 3:**
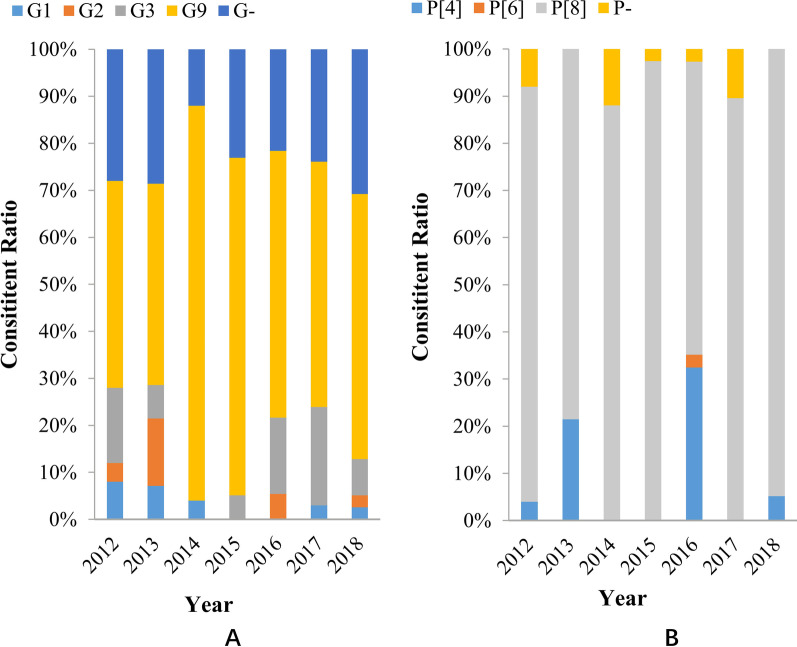
Yearly distribution of G genotypes (**A**) and P genotypes (**B**) of RVA among outpatient children with acute diarrhea in Shanghai

During the study period, 76.0% (187/246) RVA positive cases were characterized as G genotypes and the rest (24.0%, 59/246) were non-typeable. Among the definite G genotypes, G9 (58.5%, 144/246) was the most widely circulating genotype in children with acute diarrhea followed by Gx (22.8%, 56/246), G3 (12.2%, 30/246), and G1 (2.9%, 7/246) (Fig. 3A). Among P genotypes, 94.3% (232/246) RVA positive samples were successfully genotyped and 5.7% (14/246) samples werenot typed. P[8] remained to be the most frequent P genotype (86.6%, 213/246), followed by P[4] (7.3%, 18/246) and P[6] (0.4%,1/246) (Fig. 3B).

**Fig. 4:**
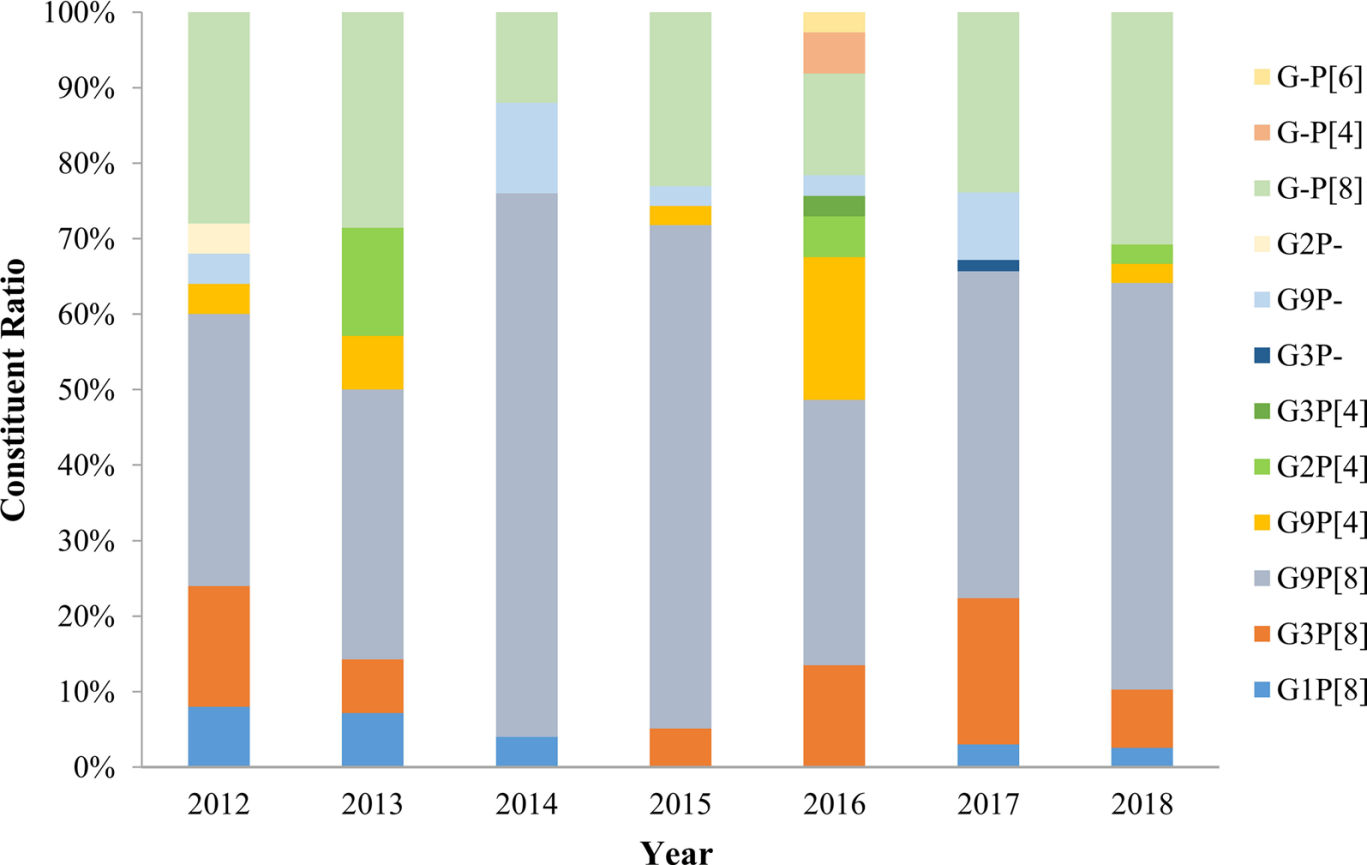
Yearly distribution of G/P combination genotypes of RVA among outpatient children with acute diarrhea in Shanghai

In G/P combinations, 12 different RVA strains were identified in this study. G9P[8] (49.2%, 121/246) was detected as the highest prevalence genotype, followed by GxP[8] (22.8%, 56/246), G3P[8] (11.4%, 28/246), G9P[x] (4.9%, 12/246), G9P[4] (4.5%, 11/246), G1P[8] (2.8%, 7/246), G2P[4] (2.0%, 5/246), GxP[4] (0.8%, 2/246), G2P[x] (0.4%, 1/246), GxP[6] (0.4%, 1/246), G3P[4] (0.4%, 1/246) and G3P[x] (0.4%, 1/246) (Fig. 4).

### Distribution of RVA genotypes among children in different age groups

**Fig. 5:**
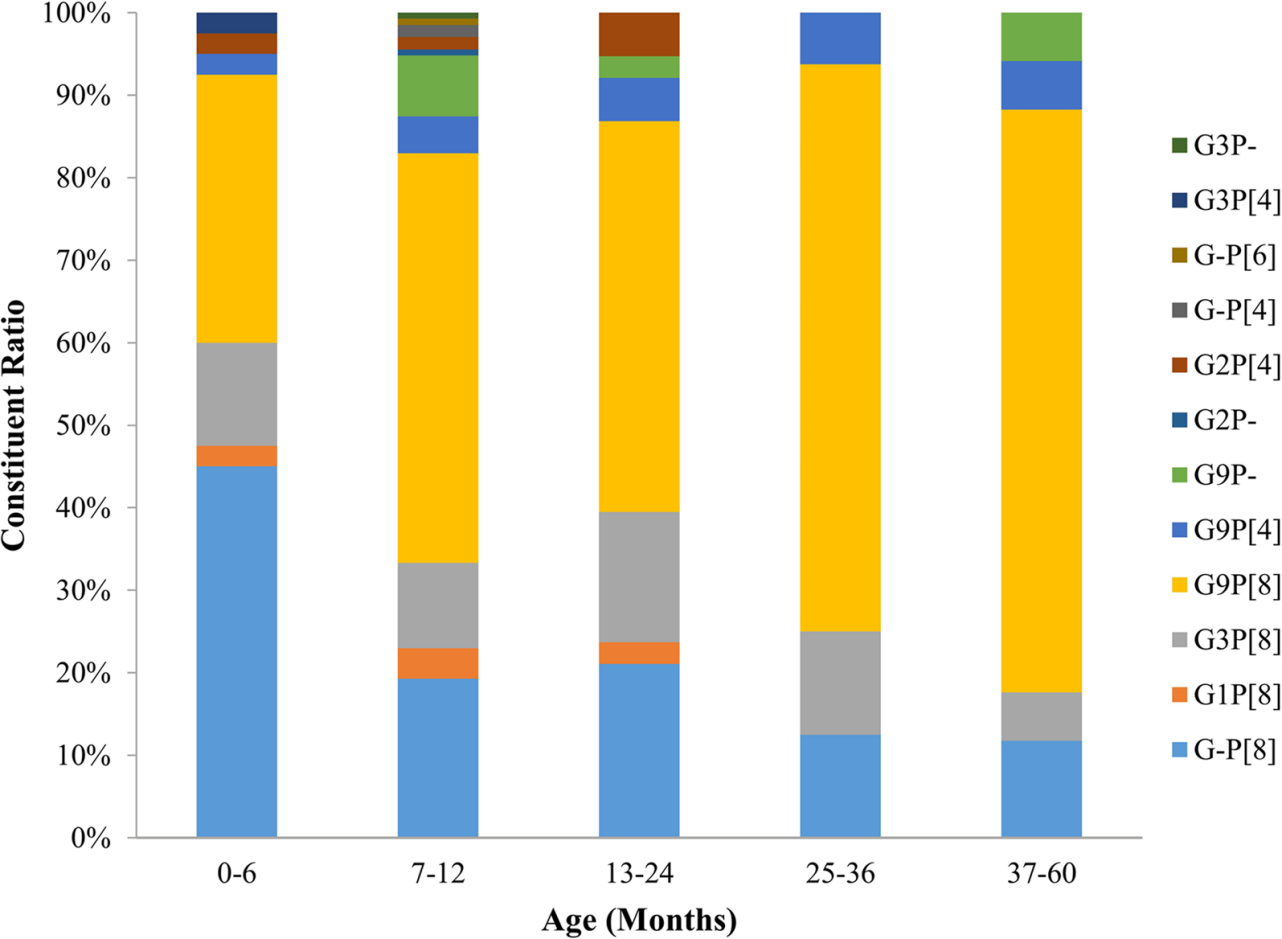
Age distribution of G/P combination genotypes of RVA among outpatient children with acute diarrhea in Shanghai

During the 7 years of the study, RVA strains infected in children aged ≤ 2 years were much more diverse compared with that in other age groups of children. In this group from 0 to 24 months, 11 different RVA strains were detected. Only 5 different RVA strains were identified in children over 2 years. GxP[8] was the most prevalent genotype in infants aged from 0 to 6 months (45.0%, 18/40) while this strain was the second prevalent genotype in children aged from 7–12 months (19.3%, 26/135), 13–24 months (21.1%, 8/38) and 37–60 months (11.8%, 2/17). G9P[8] was the most common strain in children aged from 7–12 months (49.6%, 67/135), 13–24 months (47.4%, 18/38), 25–36 months (68.8%, 11/16) and 37–60 months (70.6%, 12/17). G3P[8] was the third common strain in children aged from 0 to 6 months (12.5%, 5/40), 7 to 12 months (10.4%, 14/135) and 13 to 24 months (15.8%, 6/38) (Fig. 5).

### Co-infection of RVA with other diarrhea viruses among children

Among the 246 RVA positive samples detected in this study, 12.2% (30/246) RVA positive samples were co-infected with other diarrhea viruses. Dual infection with RV and HuCV accounted for 60.0% (18/30), followed by RVA and HAstV (26.8%, 8/30), and RVA and HAdV (10.0%, 3/30). In addition, a triple-infection (RVA co-infected with HAstV and HAdV) was also observed in this study and this child aged 3 months. Mixed infections were more likely detected in males (90.0%, 27/30) than in females (10.0%, 3/30). Furthermore, 66.7% (20/30) of the infections were mixed infections among infants aged 7–12 months (Table 1).

**Table 1:** Co-infection of RVA with other diarrhea viruses in outpatient children

Mixor single infection	Positive (n, m%)	Gender (n, m%)		Age (n, m%)				
		Male	Female	0–6	7–12	13–24	25–36	37–60
RV + HAdV + HAstV	1 (0.4)	1 (0.7)	0 (0.0)	1 (2.5)	0 (0.0)	0 (0.0)	0 (0.0)	0 (0.0)
RV + HuCV	18 (7.3)	18 (12.0)	0 (0.0)	2 (5.0)	14 (10.3)	2 (5.3)	0 (0.0)	0 (0.0)
RV + HAdV	3 (1.2)	2 (1.3)	1 (1.0)	0 (0.0)	2 (1.5)	1 (2.6)	0 (0.0)	0 (0.0)
RV + HAstV	8 (3.3)	6 (4.0)	2 (2.1)	2 (5.0)	4 (2.9)	0 (0.0)	1 (6.0)	1 (6.7)
RV	216 (87.8)	123 (82.0)	93 (96.9)	35 (87.5)	116 (85.3)	35 (92.1)	16 (94.0)	14 (93.3)
Total	246 (100.0)	150 (100.0)	96 (100.0)	40 (100.0)	136 (100.0)	38(100.0)	17 (100.0)	15 (100.0)

n: RVA positive number; m: constituent ratio of RVA infection

## Discussion

The current study provides valuable content on the prevalence and genotypes distribution of rotavirus in infants and young children with acute diarrhea from 2012 to 2018 in Shanghai. In our study, RVA is a common cause of diarrhea among children with acute diarrhea under 5 years old. This study detected a 13.6% (246 of 1814) prevalence of RVA infection in outpatient children with acute diarrhea, ranging from 9.7 to 17.4% in a fluctuation status during 2012 and 2018. This finding may be associated with the sampling method used in this study. The prevalence of RVA infection in children with acute diarrhea reported in this study is comparable with the results of Brazil from 2018 to 2019 (12%), Japan from 2015 to 2018 (19.7%), Hangzhou from 2017 to 2018 (17.3%), Chengdu from 2009 to 2014 (17.5%), Kunming from 2014 to 2015 (21.8%), Shanxi province from 2015 to 2019 (20.3%) and Beijing from 2011 to 2016 (20.8%), but lower than that in Chongqing from 2011 to 2015 (30.46%) and Southeast Asia from 2008 to 2018 (40.8%) [2, 21–28]. In the 7-year sentinel-based surveillance of RVA across China, the detection rate of RVA in high-income regions (11.2%) was similar to that in our study (13.6%) and lower than that in upper- and lower-middle-income regions (30.3% and 36.8%) [10]. We postulated that the difference in detection rate in RVA infection may be associated with the detection method used, geography, classification of economies, and status of RVA vaccination in children in the world. By comparison of the detection rate of RVA with that of norovirus referred from our previous paper, we come to the conclusion that RVA may have decreased to be the second cause of acute diarrhea in outpatient children in Shanghai [19].

Available data in this study suggest that RVA was detectable in children of all age groups, and 87.0% of RVA infection was detected in children aged ≤ 2 years. In accordance with the study in Beijing from 2011 to 2016, Kunming in 2015, and southwest in China from 2014 to 2015, RVA infection was more common in children aged from 7 to 12 months than that in children aged from 0 to 6 months [21, 22, 29]. This phenomenon could be related to the protective maternal immunity against RVA obtained in children less than 6 months [30]. However, Generally, the consensus for optimized immunization schedules to maximize the efficacy of an RVA vaccine is to vaccinate before RVA gastroenteritis occurs and before a sizeable proportion of the target population acquires natural infection [31, 32]. Taking all of this for consideration, the current immunization schedule of LLR in children who receive one dose per year for three consecutive years between the ages of 2 and 35 months may not be optimal [33]. As in previous studies in China, RVA infection in children has an obvious seasonal epidemic with the highest detection rate in the cold season [10, 32]. Meanwhile, the susceptibility of RVA between males and females was unclear in this study.

Worldwide epidemiological research on rotavirus specified that the most common G types are G1-G4, G9 and G12. However, altered trends of RVA genotype distribution have been detected over time in the same area [2, 10, 34]. Previous studies on children infected with RVA in China showed that G3 and G1 were the most prevalent G genotype before 2010. Sentinel-based surveillance reported that G9 has been becoming a robust common G genotype in children with acute diarrhea in China since 2011 [3, 10, 20]. Similar results were found in our study, G9 has played a predominant role in children infected with RVA since 2012 with a proportion of 58.5% from 2012 to 2018. However, studies reported in other Asia counties, including the Philippines from 2013 to 2015, Tunisia from 2015 to 2017, and Bangladeshi from 2014 to 2019, found that G1 was still the most predominant genotype in children infected with RVA [35–37]. According to data on the distribution of rotavirus genotypes in Japan from 2015 to 2018, G9 prevailed gradually after the introduction of RV vaccines [2]. Although the number of LLR vaccine doses administered in China has been increasing from 2000 to 2015, more and deep research is still needed on the relationship between the LLR vaccine and RVA evolution [33]. Compared with G genotypes, the prevalence of P genotypes was relatively stable in children with acute diarrhea in Shanghai. The data of our present study remain consistent with many other studies worldwide, and P[8] (86.6%) was the most frequent genotype followed by P[4] (7.3%). In contrast to studies where P[8] was the most prevalent genotype, studies in India observed P[6] as the most predominant VP4 genotype followed by globally common P[8] and P[4] genotypes.

In this study, 12 different RVA strains were identified in children with acute diarrhea. The total proportion of G9P[8], G-P[8], and G3P[8] were as high as 83.33% in all RVA strains in the study. Although RVA strains varied with the time, G9P[8] (49.2%) was the overall predominant strain in Shanghai from 2012 to 2018, which is consistent with other areas in China [21, 24, 26, 28, 38]. It's worth noting that G-P[8] (22.8%) replaced G3P[8] (11.4%) and became the second prevalent genotype in our study. The reason for a higher number of non-typeable could be due to point mutations, genetic rearrangements, reassortment events, and interspecies transmission [39–41]. Our data imply that it is necessary to update our typing method with the addition of new primers to characterize multiple genes of non-typeable RVA strains and get a more comprehensive understanding of RVA epidemiology. The circulation of G3P[8] (11.4%) and G1P[8] (2.8%) which were major epidemic strains in Shanghai before 2011, had a marked decrease during this study [20, 42]. Although LLR has been commercialized in China since 2000, several studies performed after commercialized showed that the vaccine efficacies of LLR against any genotype RVA gastroenteritis (RVGE) ranged from 35.0 to 73.3% around China [43]. Another oral human-lamb reassortant trivalent (G2-G3-G4) vaccine (LLR3) against RVGE caused by any serotype showed that the vaccine efficacies of LLR3 ranging from 56.6 to 74.0% in a phase III clinical trial. Moreover, LLR3 also showed a 70.3% efficacy against G9 sever RVGE [44]. Considering the constantly changing of RVA genotypes in children in Shanghai, LLR3 may be a more promising vaccine compared with LLR. Continuous monitoring of RVA genotype is indispensable to guide RVA vaccination strategies in children in Shanghai.

Furthermore, we also analyzed the distribution of RVA genotypes among children in different age groups. However, data on this aspect is lacking in other literature. Our research found that RVA strains detected among children in different age groups were varied. RVA strains infected in children aged ≤ 2 years were much more diverse compared with that in other age groups. Moreover, a total of 12 different RVA strains were detected in children aged from 0 to 12 months, which implicated that this age was more likely to be infected by different RVA strains. Similar results were reported in Nampula Province [45]. In addition, we also found that GxP[8] strain was the most prevalent genotype in children aged from 0 to 6 months. Due to the lack of corresponding research, the cause of this phenomenon still needs more monitoring and in-depth analysis in the following years.

Compared with our previous study from 2010 to 2011 (30.7%), the proportion of co-infection with other diarrhea viruses in this study (12.2%) showed a marked decrease (Lu et al. 2015). In line with an earlier study, dual infection of RVA and HuCV was the predominant circulating combination in children (Lu et al. 2015). Interestingly, mixed infections of diarrhea viruses were much more prevalent in males (90.0%) than in females (10.0%) in Shanghai from 2012 to 2018. However, as far as we know that no correlation between gender and the infection of diarrhea viruses was reported in previous studies. In accordance with the variety of RVA strains detected in children aged from 7 to 12 months, over half of the mixed infections also circulated in this age group. These results suggest that children aged from 7 to 12 months may be more susceptible to different RVA strains and other diarrhea viruses.

## Study limitations

Because the clinical characteristics that we could obtain from the integrated hospital information system were limited, the clinical significance of diarrhea viruses infections in children was not described in this study. RVA genotypes were detected by traditional semi-nested multiplex RT-PCR in the present study, therefore, point mutation, genetic rearrangements, and reassortment events in the evolution of RVA could not be analyzed.

## Conclusion

Our longstanding outpatient-based surveillance provided insight into trends in RVA infection. RVA was still a major cause of children with acute diarrhea in Shanghai from 2012 to 2018. A great diversity of RVA strains circulated in children with acute diarrhea with G9P[8] as the predominant genotype since 2012. In the meantime, we need to pay more attention to children aged from 7 to 12 months who were more susceptible to RVA and mixed infections with other diarrhea viruses. Overall, maintaining the rotavirus surveillance system will be important to assess the changing profile of the RVA cases as these will be the key to better management of preventive strategies such as vaccination.

## Data Availability

All data generated or analysed during this study are included in this published article.

## References

[1] Troeger C, Khalil IA, Rao PC, Cao S, Blacker BF, Ahmed T (2020). Rotavirus vaccination and the global burden of rotavirus diarrhea among children younger than 5 years. JAMA Pediatr.

[2] Hoque SA, Khandoker N, Thongprachum A, Khamrin P, Takanashi S, Okitsu S (2020). Distribution of rotavirus genotypes in Japan from 2015 to 2018: diversity in genotypes before and after the introduction of rotavirus vaccines. Vaccine.

[3] Nan X, Jinyuan W, Yan Z, Maosheng S, Hongjun L (2014). Epidemiological and clinical studies of rotavirus-induced diarrhea in China from 1994–2013. Hum Vaccin Immunother.

[4] Gupta S, Krishnan A, Sharma S, Kumar P, Aneja S, Ray P (2018). Changing pattern of prevalence, genetic diversity, and mixed infections of viruses associated with acute gastroenteritis in pediatric patients in New Delhi, India. J Med Virol.

[5] Gupta S, Chaudhary S, Bubber P, Ray P (2019). Epidemiology and genetic diversity of group A rotavirus in acute diarrhea patients in pre-vaccination era in Himachal Pradesh, India. Vaccine.

[6] Caddy S, Papa G, Borodavka A, Desselberger U (2021). Rotavirus research: 2014–2020. Virus Res.

[7] Matthijnssens J, Otto PH, Ciarlet M, Desselberger U, Van Ranst M, Johne R (2012). VP6-sequence-based cutoff values as a criterion for rotavirus species demarcation. Arch Virol.

[8] Mihalov-Kovács E, Gellért Á, Marton S, Farkas SL, Fehér E, Oldal M (2015). Candidate new rotavirus species in sheltered dogs, Hungary. Emerg Infect Dis.

[9] Bányai K, Kemenesi G, Budinski I, Földes F, Zana B, Marton S (2017). Candidate new rotavirus species in Schreiber's bats, Serbia. Infect Genet Evol.

[10] Troeger C, Khalil IA, Rao PC, Cao S, Blacker BF, Ahmed T (2018). Rotavirus vaccination and the global burden of rotavirus diarrhea among children younger than 5 years. JAMA Pediatr.

[11] Cunliffe NA, Bresee JS, Hart CA (2002). Rotavirus vaccines: development, current issues and future prospects. J Infect.

[12] Wang XY, Riewpaiboon A, von Seidlein L, Chen XB, Kilgore PE, Ma JC (2009). Potential cost-effectiveness of a rotavirus immunization program in rural China. Clin Infect Dis.

[13] Wu D, Yen C, Yin ZD, Li YX, Liu N, Liu YM (2016). The public health burden of rotavirus disease in children younger than five years and considerations for rotavirus vaccine introduction in China. Pediatr Infect Dis J.

[14] Liu N, Yen C, Fang ZY, Tate JE, Jiang B, Parashar UD (2012). Projected health impact and cost effectiveness of rotavirus vaccination among children <5 years of age in China. Vaccine.

[15] Yu J, Lai S, Geng Q, Ye C, Zhang Z, Zheng Y (2019). Prevalence of rotavirus and rapid changes in circulating rotavirus strains among children with acute diarrhea in China, 2009–2015. J Infect.

[16] Bishop R (2009). Discovery of rotavirus: implications for child health. J Gastroenterol Hepatol.

[17] Schollin Ask L (2021). Global and Swedish review of rotavirus vaccines showed considerable reductions in morbidity and mortality. Acta Paediatr.

[18] Sadiq A, Bostan N, Yinda KC, Naseem S, Sattar S (2018). Rotavirus: genetics, pathogenesis and vaccine advances. Rev Med Virol.

[19] Lu L, Zhong H, Xu M, Su L, Cao L, Jia R (2019). Genetic diversity and epidemiology of Genogroup II noroviruses in children with acute sporadic gastroenteritis in Shanghai, China, 2012–2017. BMC Infect Dis.

[20] Lu L, Jia R, Zhong H, Xu M, Su L, Cao L (2015). Molecular characterization and multiple infections of rotavirus, norovirus, sapovirus, astrovirus and adenovirus in outpatients with sporadic gastroenteritis in Shanghai, China, 2010–2011. Arch Virol.

[21] Tian Y, Chughtai AA, Gao Z, Yan H, Chen Y, Liu B (2018). Prevalence and genotypes of group A rotavirus among outpatient children under five years old with diarrhea in Beijing, China, 2011–2016. BMC Infect Dis.

[22] Kang Y, Cai Y (2018). Epidemiology and genetic diversity of rotavirus in Kunming, China, in 2015. Intervirology.

[23] Chen YH, Chen F, Zhou T, Chen JY, Zheng TL, Xu X (2018). Prevalence and clinical profile of rotavirus A infection among diarrhoeal children and phylogenetic analysis with vaccine strains in Chengdu, West China, 2009–2014. Trop Med Int Health.

[24] Zeng Y, Li T, Zhao B, Lai F, Tang X, Qiao Y (2019). Molecular epidemiology of group A rotavirus in outpatient diarrhea infants and children in Chongqing, China, 2011–2015. J Med Virol.

[25] Gutierrez MB, Fialho AM, Maranhão AG, Malta FC, Andrade JDSR, Assis RMS (2020). Rotavirus A in Brazil: molecular epidemiology and surveillance during 2018–2019. Pathogens.

[26] Li W, Xiang W, Li C, Xu J, Zhou D, Shang S (2020). Molecular epidemiology of rotavirus A and adenovirus among children with acute diarrhea in Hangzhou. China Gut Pathog.

[27] Lestari FB, Vongpunsawad S, Wanlapakorn N, Poovorawan Y (2020). Rotavirus infection in children in Southeast Asia 2008–2018: disease burden, genotype distribution, seasonality, and vaccination. J Biomed Sci.

[28] Zhao L, Shi X, Meng D, Guo J, Li Y, Liang L (2021). Prevalence and genotype distribution of group A rotavirus circulating in Shanxi Province, China during 2015–2019. BMC Infect Dis.

[29] Zhang S, Yin J, Yang J, Tian L, Li D, Zhang Q (2017). Epidemiology and genetic diversity of group A rotavirus in acute diarrhea patients in pre-vaccination era in southwest China. J Med Virol.

[30] Levine MM, Robins-Browne RM (2012). Factors that explain excretion of enteric pathogens by persons without diarrhea. Clin Infect Dis.

[31] Bresee JS, Hummelman E, Nelson EA, Glass RI (2005). Rotavirus in Asia: the value of surveillance for informing decisions about the introduction of new vaccines. J Infect Dis.

[32] Wang XY, Xu ZY, von Seidlein L, Zhang YL, Zhao SJ, Hao ZY (2005). Incidence of diarrhea caused by rotavirus infections in rural Zhengding, China: prospective, population-based surveillance. J Infect Dis.

[33] Fu C, Dong Z, Shen J, Yang Z, Liao Y, Hu W (2018). Rotavirus gastroenteritis infection among children vaccinated and unvaccinated with rotavirus vaccine in southern china: a population-based assessment. JAMA Netw Open.

[34] Angkeabos N, Rin E, Vichit O, Chea C, Tech N, Payne DC (2018). Pediatric hospitalizations attributable to rotavirus gastroenteritis among Cambodian children: seven years of active surveillance, 2010–2016. Vaccine.

[35] Bonifacio J, Lupisan S, Roque V, Ducusin MJ, Grabovac V, Batmunkh N (2018). Molecular characterization of rotavirus diarrhea among children aged under five years in the Philippines, 2013–2015. Vaccine.

[36] Bennour H, Fodha I, Bouazizi A, Ben Hamida-Rebaï M, Jerbi A, Fredj MBH (2019). Molecular characterization of group A rotavirus among children aged under 5 years in Tunisia, 2015–2017. J Med Microbiol.

[37] Dey SK, Sharif N, Sarkar OS, Sarkar MK, Talukder AA, Phan T (2020). Molecular epidemiology and surveillance of circulating rotavirus among children with gastroenteritis in Bangladesh during 2014–2019. PLoS ONE.

[38] Kuang X, Gong X, Zhang X, Pan H, Teng Z (2020). Genetic diversity of group A rotavirus in acute gastroenteritis outpatients in Shanghai from 2017 to 2018. BMC Infect Dis.

[39] Iturriza-Gómara M, Kang G, Gray J (2004). Rotavirus genotyping: keeping up with an evolving population of human rotaviruses. J Clin Virol.

[40] Sharma S, Paul VK, Bhan MK, Ray P (2009). Genomic characterization of nontypeable rotaviruses and detection of a rare G8 strain in Delhi. India J Clin Microbiol.

[41] Iturriza-Gómara M, Dallman T, Bányai K, Böttiger B, Buesa J, Diedrich S (2011). Rotavirus genotypes co-circulating in Europe between 2006 and 2009 as determined by EuroRotaNet, a pan-European collaborative strain surveillance network. Epidemiol Infect.

[42] Xu J, Yang Y, Sun J, Ding Y, Su L, Fang Z (2009). Molecular epidemiology of rotavirus infections among children hospitalized for acute gastroenteritis in Shanghai, China, 2001 through 2005. J Clin Virol.

[43] Zhen SS, Li Y, Wang SM, Zhang XJ, Hao ZY, Chen Y (2015). Effectiveness of the live attenuated rotavirus vaccine produced by a domestic manufacturer in China studied using a population-based case-control design. Emerg Microbes Infect..

[44] Xia S, Du J, Su J, Liu Y, Huang L, Yu Q (2020). Efficacy, immunogenicity and safety of a trivalent live human–lamb reassortant rotavirus vaccine (LLR3) in healthy Chinese infants: a randomized, double-blind, placebo-controlled trial. Vaccine.

[45] Chissaque A, Bauhofer AFL, Cossa-Moiane I, Sitoe E, Munlela B (2021). Rotavirus A infection in pre- and post-vaccine period: risk factors, genotypes distribution by vaccination status and age of children in Nampula Province, Northern Mozambique (2015–2019). PLoS ONE.

